# Exploring clusters of defense styles, psychiatric symptoms and academic achievements among medical students: a cross-sectional study in Pakistan

**DOI:** 10.1186/s13104-018-3876-6

**Published:** 2018-11-01

**Authors:** Ahmed Waqas, Sadiq Naveed, Kapil Kiran Aedma, Maryam Tariq, Tayyaba Afzaal

**Affiliations:** 1 CMH Lahore Medical College & Institute of Dentistry, Lahore Cantt, Pakistan; 2 Human Development Research Foundation, Rawalpindi, Pakistan; 3 KVC Hospitals, Kansas City, KS USA; 4 Dow University of Health Sciences, Karachi, Pakistan; 5 Government College University, Lahore, Pakistan

**Keywords:** Ego defense mechanisms, Defense styles, DSQ-40, Defense style questionnaire, Anxiety, Depression, Medical students, Pakistan, Academic performance, Medical education

## Abstract

**Objective:**

The clusters of participants with a homogeneous psychological make-up can be identified using sophisticated machine learning techniques such as the two-step clustering algorithm. It can also help us to identify the synergistic and additive effects of a range of psychometric variables. The identification of synergistic effect of this clustering of defense mechanism has significant practical implications as they share a certain variance. This study aims to identify the clusters of ego defenses and their relationship with academic performance and mental health outcome in medical students.

**Results:**

The high achievers scored higher on mature and neurotic defense styles and lower on immature than their counter parts. A higher proportion of medical students in high achievers group had normal scores on depressive symptoms than low achievers. While a majority among low achievers suffered from severe anxiety levels than high achievers group. High achievers scored higher on sublimation, humor, anticipation, suppression, pseudo-altruism, idealization, reaction formation, autistic fantasy, denial, and rationalization.

**Electronic supplementary material:**

The online version of this article (10.1186/s13104-018-3876-6) contains supplementary material, which is available to authorized users.

## Introduction

Ego defenses are a set of unconscious cognitive processes that help an individual ward off unwarranted anxiety [1]. These defenses tend to protect an individual’s biopsychic integrity, and depending on their level of maturity; contribute significantly to personal growth and development [2]. In this context, George Vaillant proposed a hierarchy of defenses in which mature defenses were associated with better adaptive functioning than their neurotic and immature counterparts [3]. Given the positive correlation of mature defense mechanisms with higher level of functioning, better problem-solving and coping strategies, a higher academic performance is expected [4]. This relationship was also tested in our previous paper, employing multivariate regression technique (Additional file 1) [5].

Stress-illness relationship can be explained by underlying defense mechanisms, where the clustering of immature ego defenses can lead to psychological impairment specially anxiety and depression. Several studies in general population have linked psychiatric morbidities including depression with maladaptive defense mechanism [6–8]. Since medical students are more prone to develop anxiety and depression than the general population, as shown by studies conducted in the region [9], it is imperative to elucidate the additive/synergistic effects of these underlying defense mechanism on their psychological health.

It is opined that different health related behaviors especially psychological defenses do not exist in isolation, rather exist in clusters that are not uniformly distributed among population [2–4]. Recent research has indicated that this clustering of unhealthy behaviors act synergistically, leading to more detrimental effects than their isolated effects [9]. Despite a range of several hypothetical frameworks analyzing academic performance, there is a significant dearth of literature identifying clusters of ego defenses among medical students affecting their academic performance and mental health outcomes. These clusters of participants with a homogeneous psychological make-up and synergistic effects of a range of psychometric variables, can be identified using sophisticated analytical techniques such as the two-step clustering algorithm.

The identification of this clustering of defense mechanism has significant practical implications as they share a certain variance. Changing the pattern of these unconscious mechanisms through intervention programs and assisting medical students to acquire better insight of their behaviors, can encourage use of mature defenses and better functioning.

## Main text

### Methods

This cross-sectional study was conducted from December 1, 2014 to January 15, 2015, at two privately financed medical schools namely: the CMH Lahore Medical College (CMH LMC) and the Fatima Memorial College of Medicine and Dentistry, both in Lahore, Pakistan. Ethical approval was sought and obtained from the Ethical review committee of the CMH Lahore Medical College, Lahore Cantt, Pakistan. A total of 500 medical students were approached conveniently during their class hours. Written informed consent was obtained from all respondents who were assured anonymity and exclusive use of data for scholarly purposes. The questionnaire proforma comprised of a section recording demographics and grades obtained on their annual examination, the Hospital Anxiety and Depression scale (HADS) and the defense style questionnaire-40 (DSQ-40), published in our previous study [7].

The HADS is a 14-item self-administered questionnaire that assesses the respondents’ levels of anxiety and depression. It has shown excellent reliability statistics among Pakistani medical students in previous validation studies  [10]. The scores on the depression and anxiety subscales are calculated by summing the respective items, yielding a score ranging from 0 to 21. Moreover, these scores also be categorized into normal (0 to 7), borderline anxious/depressed (8–10), and severely anxious/depressed (11–21).

The DSQ-40 assesses use of ego defense mechanisms among the respondents, broadly classifying these into three hierarchies: mature, neurotic and immature defense styles [11]. These defense styles comprise of defense mechanisms classified by Andrews as: “(a) four mature: sublimation, humor, anticipation, and suppression; (b) four neurotic: undoing, pseudo-altruism, idealization, and reaction formation; and (c) twelve immature: projection, passive aggression, acting out, isolation, devaluation, autistic fantasy, denial, displacement, dissociation, splitting, rationalization, and somatization.” Each defense mechanism is covered by 2 items in the DSQ-40 [11]. The DSQ-40 exhibited a good internal consistency in the present sample with a Cronbach’s alpha of 0.75.

All data were analyzed with SPSS v.20. Frequencies (%) were calculated for categorical variables and mean (SD) for quantitative variables. Thereafter, two step clustering analysis was run to identify clusters of students with a homogenous groups of ego defense styles, comparable academic performance and reporting similar severity levels of anxiety and depressive symptomatology. Individual ego defense mechanisms were introduced as evaluation fields. Quintessentially, the two step clustering algorithm is a scalable cluster analysis that is capable of analyzing both continuous and categorical variables by utilizing the model-based distance measure and automatically retains the optimal number of clusters [12]. All continuous variables were standardized into z-scores before running the cluster analysis [12]. Log-likelihood based distance measure was employed with the best clustering solution based on the Akaike Information Criterion (AIC) [13]. The silhouette measure of cohesion and separation was used to evaluate the goodness of fit of the clustering solution, where a value > 0.50 represents a good solution [14].

### Results

A total of 409 (response rate = 81.80%) medical students responded to the survey, with 253 (61.90%) females and 156 (38.10%) males. A total of 258 medical students (63.1%) were enrolled in pre-clinical years while 150 (36.70%) in clinical years. There were a total 129 (31.5%) belonging to middle income groups and 280 (68.5%) from high income groups. Mean age (SD) of the respondents was 19.86 (1.33) years and mean percentage of marks obtained 75.61 (9.13) years. A total of 83 (20.30%) were borderline and 35 (8.6%) severely depressed while 131 (32%) were borderline and 150 (36.70%) were severely anxious. Their mean scores on the anxiety subscale were 9.5 (3.9) and 5.8 (3.12) on the depression subscale. Detailed scoring of DSQ-40 and HADS scale in present sample can be read in a previous publication [5].

**Fig. 1:**
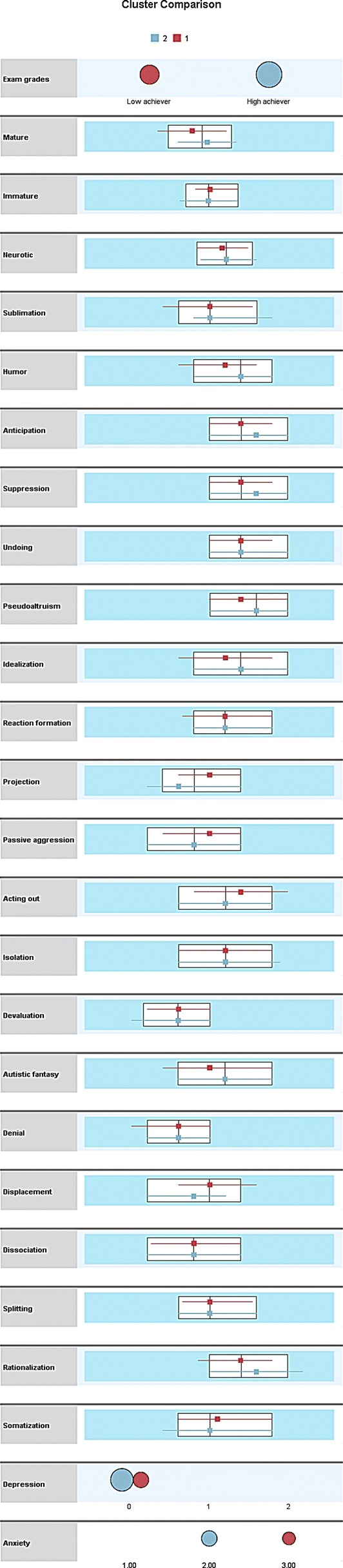
Graphical presentation of clusters of medical students as identified in two step clustering analysis

Firstly, clusters of students based on their academic performance and psychiatric symptomatology (anxiety and depression) were explored. It yielded a satisfactory cluster model with an acceptable value of silhouette measure of cohesion and separation (0.6). The ratio of sizes of clusters was 1.81, presenting two clusters: low academic achievers (n = 243, 64.50%) and high academic achievers (n = 134, 35.50%), based on their academic grades (≤ 70% vs > 70%. The high achievers scored higher on mature and neurotic defense styles and lower on immature than their counter parts. A higher proportion of medical students in high achievers group had normal scores on depressive symptoms than low achievers. While a majority among low achievers suffered from severe anxiety levels than high achievers group. Results of individual defense mechanisms are presented in Table 1 and Fig. 1. High achievers scored higher on sublimation, humor, anticipation, suppression, pseudo-altruism, idealization, reaction formation, autistic fantasy, denial, and rationalization. Participants in the high achievers group were younger (19.38 years vs 20.60 years, females (Chi square = 5.63, P = 0.018) belonging to pre-clinical years (Chi square = 39.25, P < 0.001).

**Table 1:** Detailed tabulated presentation of clusters of medical students

Variable	High achievers	Low achievers
Size	243	134
Exam grades	High achievers (100%)	Low achievers (100%)
Mature	5.71	5.43
Immature	4.92	5.09
Neurotic	5.92	5.79
Sublimation	5.47	5.01
Humor	5.77	5.52
Anticipation	6.19	6.09
Suppression	6.19	6.09
Undoing	5.99	6.06
Pseudo-altruism	6.28	5.98
Idealization	5.91	5.5
Reaction formation	5.5	5.32
Projection	4.5	5.0
Passive aggression	4.58	4.84
Acting out	5.43	5.88
Isolation	5.51	5.54
Devaluation	3.95	4.15
Autistic fantasy	5.33	5.29
Denial	4.16	4.05
Displacement	4.3	5.21
Dissociation	4.48	4.56
Splitting	5.22	5.31
Rationalization	6.34	5.78
Somatization	5.18	5.41
Depression	Normal (76.5%)	Normal (65.7%)
Anxiety	Borderline (36.2%)	Severe (44.8%)

### Discussion

We identified two distinct clusters in our study namely high academic achievers and low academic achievers after integrating the defense styles with academic performance. The high achievers predominantly employed mature and neurotic defense styles whereas the low achievers employed immature defense mechanisms to a relatively greater extent.

Our findings are consistent with MacCann et al., showing mature defense mechanisms to be associated with better academic performance than immature defenses [6]. The clustering of increased academic productivity with mature defenses can in turn be explained by a number of factors; firstly, Grebot et al. found the association of mature defenses with problem focused coping and immature defenses with avoidance coping [5]. Secondly, some predictors of good academic performance amongst constructive personality traits of self-competence, self-esteem, proficiency, sociability and confidence are strongly linked to the use of mature defenses; in contrast trouble indicators of recklessness, egocentricity and ambiguous thinking style were associated with the use of immature defense mechanism [15–18]. Thirdly, the use of mature defenses is an indicator of later positive adjustment in young adults. Finally a higher IQ has generally been linked with the use of mature defenses associated with a higher academic productivity in this cluster [19, 20].

Our findings also highlight the clustering of neurotic defenses among high achievers despite their association with maladaptive outcomes in literature [21]. Multiple studies replicate the findings of pseudo-altruism, anticipation and rationalization as one of the most commonly employed defense mechanisms among medical students [22, 23]. In terms of individual defenses, our findings reveal inquisitive associations between the defense mechanisms of pseudo altruism, autistic fantasy, rationalization and idealization among high achieving medical students. The adaptive nature of pseudo-altruism has also been highlighted by Vaillant [21], citing that the greater use of this mechanism is associated with greater accountability towards patients which in turn increases academic productivity. Our previous research also highlights an association of rationalization with higher grades and its employment to deal with the academic stressors. La Cour reported that use of rationalization aids medical students in dealing with the forbidding veracities at their workplace [5, 23]. This finding is in contrast with Negrii who reported a greater use of rationalization in students with low grades [2].

The high achieving cluster in our research also made significant use of idealization. Cramer labeled this as a “healthy” defense and a part of a normal maturational process [24]. An integral part of this defense is to maintain self-worth by internalizing the admired characters into self-representation. Hence, it can be postulated that it acts as a driving force in the lives of medical students making them more diligent and committed towards medical field which is then reflected in their academic performance.

The cluster of low achievers scored significantly higher on projection, passive aggression, acting out, displacement and somatization. These are highly maladaptive, reality-distorting (projection) or minimizing (displacement) defenses, disabling a person to effectively resolute conflicts of conscience, emotions and interpersonal relationships [21]. One of the functions of immature defenses is to mitigate the intimidating self-esteem [25]. Given the well-researched correlation of low self-esteem with poor academic performance, the clustering of these defenses with low academic output can be well understood. The increased projection in this cluster has previously been explained by Cramer et al. [26], who suggested a relationship between EDM and ego level as a function of IQ; a stronger use of projection and denial with low IQ that maintains higher ego levels.

Low achievers also significantly scored higher on somatization, in consonance with earlier literature revealing poor academic performance in high- somatizing school children [27]. Similarly significant use of passive aggression, displacement and acting out can be explained as a shielding mechanism against confidence loss in this cluster [28].

Our previous study highlighted the association of lower anxiety and depression scores with higher academic performance [5], however using the sophisticated technique of cluster analysis on the same sample yielded two groups scoring low on depression. The low achievers were severely anxious while the high achievers maintained moderate anxiety levels. Our findings are in perfect line with the Yerkes Dodson’s curve which connotes optimum performance with moderate level of anxiety while impaired performance is linked to both high and low levels of anxiety [29]. These results in part contradict the earlier research findings associating low anxiety and depression with mature defenses and higher depression and anxiety with immature defenses [7, 30]. It is interesting to note that the low achievers scored low on the depression severity despite the notion that the use of immature defense mechanisms is linked with depression [31, 32], which can be explained by clustering of some mature defense mechanisms [21].

### Conclusion

The use of clustering mechanism revealed distinct uses of defense styles among the high and low achievers enrolled in medical schools in Pakistan. A pragmatic appraisal of this clustering pattern would enable integrated behavior techniques, designed to enhance the adaptation of “healthier” defenses.

## Limitations

The generalizability of the results of this study are limited by its cross-sectional nature, potential recall bias and inclusion of participants at two Pakistani medical schools only. Although the data is 3.5 years old, it is still representative of the norms in present time [19, 33].

## Additional file

**Additional file 1.** This data file contains all the data reported in the study.

## Abbreviations

DSQ-40: defense style questionnaire (40 items)
HADS: hospital anxiety and depression scale
EDM: ego defense mechanism
